# Differences in implementation of family focused practice in hospitals: a cross-sectional study

**DOI:** 10.1186/s13033-018-0256-5

**Published:** 2018-12-19

**Authors:** Bjørg Eva Skogøy, Darryl Maybery, Torleif Ruud, Knut Sørgaard, Gro Christensen Peck, Elin Kufås, Kristin Stavnes, Eivind Thorsen, Terje Ogden

**Affiliations:** 1 Nordland Hospital Trust, Kløveråsveien 1, 8092 Bodø, Norway; 2 The Faculty of Health Science, UiT The Arctic University of Norway, Box 6050, 9037 Tromsø, Norway; 3 Monash University Department of Rural Health, Box 973, Moe, VIC 3825 Australia; 4 Department for Research and Development, Mental Health Services, Akershus University Hospital, Box 1000, 1478 Lørenskog, Norway; 5 Institute of Clinical Medicine, University of Oslo, Blindern, Box 1171, 0318 Oslo, Norway; 6 Vestre Viken Hospital Trust, Box 800, 3004 Drammen, Norway; 7 Stavanger University Hospital, Box 8100, 4068 Stavanger, Norway; 8 BarnsBeste (Children’s Best Interests) - National Competence Network for Children as Next of Kin, Sørlandet Hospital Trust, Box 416, 4604 Kristiansand, Norway; 9 Norwegian Centre for Child Behavioural Development, Unirand, Majorstuen, Box 7053 0368 Oslo, Norway; 10 Institute of Psychology, University of Oslo, Blindern, Box 1171, 0318 Oslo, Norway

**Keywords:** Policy, Law, Family focused practice, Hospitals, Children as next of kin, Children of ill parents, Parental illness, Child responsible personnel

## Abstract

**Background:**

Changes in Norwegian law and health policy require all health professionals to help safeguard the provision of information and follow-up for the children of parents with mental or physical illness, or substance abuse problems, to decrease their risk of psychosocial problems. There is a lack of knowledge on how the national changes have been received by hospital-based health professionals, and if they have led to an increase in family focused practice.

**Methods:**

This cross-sectional study examined the adherence of health professionals’ (*N* = 280) in five hospitals to new guidelines for family focused practice, using a translated and generic version of Family Focused Mental Health Practice Questionnaire.

**Results:**

Overall, health professionals scored high on knowledge and skills, and were confident in working with families and children, but reported moderate levels of family support and referrals. Comparison of the five hospitals showed significant differences in terms of workplace support, knowledge and skills and family support. The smallest hospital had less workplace support and less knowledge and skills but scored medium on family support. The two largest hospitals scored highest on family support, but with significant differences on parents refusing to have conversations with children.

**Conclusions:**

Differences in implementation of family focused practice highlight the need to tailor improvement strategies to specific barriers at the different hospitals. The use of implementation theories and improvement strategies could promote full implementation, where all families and children in need were identified and had access to family support.

*Trial registration* The study is approved by the Regional Committee on Medical and Health Research Ethics South-East Q5 37 (reg. no. 2012/1176) and by the Privacy Ombudsman.

## Background

Norway [1], Finland [2] and Sweden [3, 4] now require all health professionals to encourage support for children of parents with all types of illnesses. The regulations deriving from the 2010 law in Norway require all health professionals to; (a) register dependent children in the patient’s health record, (b) have conversations with the parent about children’s need for information and support, (c) offer help in family information sharing and conversations with children, (d) ensure that children can visit parents at the hospital, (e) assess children’s and the family’s needs, and (f) gain parents’ consent to cooperate with other services in establishing necessary support [5].

These regulations are in line with international recommendations to include family focused, family centred, family sensitive, family oriented, family based, family inclusive or child cantered practices to support the children of parents ill with mental health [6–14], substance abuse [15, 16] or physical illness [17–19].

These changes in law are based on research evidence. Increasing parenting skills and improving young people’s knowledge and resilience are key elements in 13 individual, group and family interventions that were recently meta-analysed, showing that these factors reduce by 40% children’s risk of developing the same mental illness as their parents [20]. In physical health, a systematic review of 19 psychosocial interventions for families with parental cancer also showed most to be helpful, with improvements in quality of life and mental health or distress [21]. Prevention of mental illness in families is a prominent feature of recent health policies that has been found to work in mental health prevention practices.

Family focused practice supports the whole family unit, both the parents with an illness, and the children [6], and it has been suggested that it includes a continuum of practices [22], with core elements such as; family care planning, goal-setting, liaison between families and services, instrumental, emotional and social support, assessment of family members, psychoeducation, and a coordinated system of care between families and services [6].

Many children are affected by parental illness. A recent systematic review of 9 studies showed parent prevalence among patients in adult psychiatric services to range from 12.2 to 45.0% [23]. In Norway, parent prevalence is estimated to be 10.4–23.1% for severe and moderate mental disorders [24], with severe alcohol use disorder estimated to affect 2.7% of Norwegian children [24]. Physical illnesses such as cancer are estimated to affect 3.1% of Norwegian children (0–18 years), and 8.4% of young adults (19–25 years) [25]. Internationally, approximately 10% of children are estimated to have a parent with a chronic medical condition such as cancer and multiple sclerosis [26]. It should be noted that estimates of parental illness vary in whether they include severe, moderate or broader categories of illness.

Researchers have noted numerous barriers to implementing family focused practices. These include differences across countries, organisational factors such as lack of resources and inadequate procedures, professional background, cultural and educational factors such as health professionals’ attitudes and lack of expertise, lack of cooperation, and access to families [27–32]. More generally, it is recommended to tailor implementation strategies to different practice settings, and groups of practitioners to overcome implementation barriers [33, 34].

The Active Implementation Framework (AIF) [35–37] describes four implementation stages. In the exploration stage, needs are assessed, fit and feasibility of the intervention model is examined, stakeholders are involved, and an implementation plan is made. In the installation stage, implementation support is developed alongside necessary structural and instrumental changes. In the initial implementation stage, new services for families and children are delivered. At this stage it is important to use data to drive decision-making, alongside a rapid-cycle problem-solving approach to make necessary improvements. The final stage is full implementation, where systems and organisational changes are established and become part of sustained routine practice. This article pertains to the full implementation stage.

Implementation research shows that changing daily practice by introducing evidence and clinical guidelines requires comprehensive approaches at different levels. It is recommended to tailor interventions to specific settings and target groups [33]. Plans for change should be based on characteristics of the evidence or guidelines and on known barriers and facilitators of change [38–40].

Leadership plays a critical role in creating organisational readiness for change [41]. Leaders who can inspire and motivate employees have been found to predict implementation of innovative practice [42], to be associated with innovation climate and more positive staff attitudes to the adoption of evidence-based practice [43], and to be a predictor of implementation satisfaction [44]. Large organisations often have more resources than small ones, such as knowledge and skills for innovative practice and role specialisation [44–46].

Change agents [47] or champions [39, 48]) have been found to play an important role in innovative practice, and without their contributions it is less likely that new practice will be implemented [46]. Norwegian hospitals must comply with the law by having child responsible personnel (CRP). They have a common responsibility to support change across all hospitals, and to promote and coordinate support given by health professionals to parents as patients and their children. Being a CRP comes in addition to the health professional’s ordinary work and with no extra remuneration. Some of them have participated in a 2 by 2 days training programme piloted by the national competency network and/or taken an e-learning programme (http://www.barnsbeste.no). Others have been offered training and supervision by their hospital.

## Aims

This study examines the level of family focused practice in Norway following the 2010 changes according to the law. The study is part of a large multicentre study (The CHIP-study) [49] of patients’, partners’, and children‘s satisfaction with the implementation of the changes to the law, and of the follow up on children´s needs when parents have a mental illness, a serious physical illness, or substance abuse. The first aim was to describe the type and extent of family focused practice in the five hospitals taking part in the study. The second aim was to explore any differences in family focused practices between the five hospitals.

## Methods

### Design

This was an exploratory cross-sectional study.

### Context

The five hospitals in this study serve 34% of the total Norwegian population of 5.2 million. To get maximum diversity we included five hospitals of different sizes, from three regions across Norway, including both rural and urban areas. Hospital 1 (H1) serves 136,000 inhabitants, and the others serve 290,000 (H2), 358,000 (H3), 480,000 (H4) and 493,000 (H5) inhabitants respectively [50]. H1 is the smallest hospital and provides health services to a large rural area, and H3 and H5 are university hospitals. Each hospital had appointed CRPs to support and systematise the work. Four of the hospitals had appointed a hospital coordinator (H-CRP), with H1 having coordinators at a lower level. Hospital 5 was the only hospital with a full-time H-CRP. There were from 21 to 45 CRPs per 100,000 inhabitants served, with the two largest hospitals having a smaller number of CRPs per 100,000 (H1: 39, H2: 45, H3: 41, H4: 21, H5: 24).

### Sample

The 280 health professionals participating in this study were recruited from stratified, randomly selected outpatient and inpatient units for physical illness (cancer and neurological illness), mental illness and substance abuse in the five hospitals. A group of CRPs, (n = 104, 72% response rate) with one CRP per unit. A second group was recruited from other clinicians (C) treating patients who were recruited for the larger part of the CHIP-study (n = 176, 52% response rate). Among them, 32 were also CRP who was subsequently added to the CRP above.

There were significant differences between the hospitals regarding professional background and age. Participants at H5 were on average 6 years younger than at H2. More social workers participated from H4 than from H3, more physicians from H2 than from H3, and more others (e.g. family therapists, physical therapists, occupational therapists, hospital chaplains and nurse assistants) from H1 than from H5. There was no significant difference between the hospitals in the number of CRPs, or whether health professionals had received specific training after changes to the law, see Table 1. The online questionnaire was designed to avoid missing values when completing the FFPQ.

**Table 1:** Differences across Hospitals of participants’ background and role (N = 280)

	Total	H1 (n = 73)	H2 (n = 41	H3 (n = 43)	H4 (n = 65)	H5 (n = 58)	*p*
Gender							
Women (%)	224 (80)	62 (84.9)	28 (68.3)	33 (76.7)	52 (80.0)	49 (84.5)	.228
Men (%)	56 (20)	11 (15.1)	13 (31.7)	10 (23.3)	13 (20.0)	9 (15.5)	.228
Age (SD)	45.4 (10.2)	45.0 (9.5)	49.5 (8.5)	44.2 (10.1)	46.2 (10.7)	43.1 (10.7)	.029*
Length of exp. (SD)	18 (10.1)	17 (9.9)	21 (10.7)	18 (9.8)	17 (10.3)	15 (9.7)	.109
Years in post (SD)	6.1 (5.6)	6.3 (5.6)	8.2 (6.2)	5.5 (5.7)	6.4 (5.3)	4.6 (5.2)	.118
Profession							
Nurse (%)	101 (36.1)	29 (39.7)	17 (41.5)	17 (39.5)	16 (24.6)	22 (37.9)	.292
Social worker (%)	42 (15.0)	6 (8.2)	3 (7.3)	6 (14.0)	17 (26.2)	10 (17.2)	.025*
Psychologist (%)	71 (25. 4)	15 (20.5)	5 (12.2)	13 (30.2)	17 (26.2)	21 (36.2)	.066
Physician (%)	32 (11.4)	9 (12.3)	9 (22.0)	0 (0)	10 (15.4)	4 (6.9)	.015*
Other (%)	34 (12.1)	14 (19.2)	7 (17.1)	7 (16.3)	5 (7.7)	4 (1.7)	.016*
Role							
CRP (%)	136 (48.6)	33 (45.2)	21 (51.2)	27 (62.8))	28 (43.1))	27 (46.8)	.308
C (%)	144 (51.4)	40 (54.8)	20 (48.8)	16 (37.2)	37 (56.2)	31 (53.4)	.308
Specific training (SD)	1.04 (.85)	1.07 (.82)	1.05 (.90)	1.16 (.80)	1.08 (.80)	.84 (.83)	.385

* p < .05

### Data collection

The data were collected from June 2013 to December 2014. Health professionals (child responsible personnel and other clinicians) received an e-mail invitation with reminders. Link and password to the web-based version of the FFPQ [51] were distributed after confirmation of participation.

### Measure

The measure employed in this study was adapted from the Family Focused Mental Health Questionnaire [51]. The questionnaire has been used regarding family focused practice in relation to parental mental health problems in Australia [31, 52, 53], Ireland [27] and Thailand [54]. The 49-item measure with 17 subscales employs a seven-point Likert Scale. Scores ranged from 1 to 7, from *strongly disagree* = 1 to *strongly agree* = 7, and in addition *not applicable* (N/A).

The measure was translated into Norwegian and made generic, to focus health professionals’ work with parents affected by all kinds of illnesses (not solely mental health), which makes it possible to use the same questionnaire also in somatic clinics and in substance abuse clinics. The translation was made by two persons separately, and differences were discussed with three colleges/supervisors to reach consensus. Back-translation was conducted by a native English-speaking person, followed by further discussions with the authors before finalising the Norwegian version.

Content validity of the items in the questionnaire was discussed with a sample of experts in this area, and the clarity of the questions and layout was tested in a pilot study with health professionals and user consultants. The main changes from the original questionnaire were that *mental illness* were replaced by *illness*, *mental health workers* were replaced by *health professionals*, and the explanation before the questionnaire stated that the aim was *to explore family focused practice within all types of illnesses* (mental illness, physical illness and substance abuse), as required by the Norwegian changes to the law.

Reliability of the measure was analysed using Cronbach’s alpha reliability analysis, using SPSS (version 24). Three items were removed from subscales, to increase reliability on the training, confidence and family support subscales. Reliability of the subscales ranged from .17 to .80, with seven scales scoring under .60. In this article, we report only the ten subscales that scored over .60, with five of them scoring over .70 (see Table 2).

**Table 2:** Descriptive statistics of family focused practice subscales, definitions, and reliability (Cronbach’s alphas) (N = 280)

Subscale	Subscale definition	α	*M (SD)*
Workplace support	The workplace provides support (e.g. supervision) for family focused practice.	.67	4.52 (1.54)
Co-worker support	The support from other workers regarding family focused work	.62	5.08 (1.13)
Time family work	Time or workload constraints regarding family focused practice	.80	4.50 (1.45)
Service available	There are programmes to refer families to	.62	4.85 (1.34)
Knowledge skills	Worker skill and knowledge regarding impact of parental mental illness on children	.76	4.93 (1.00)
Connectedness	Workers’ assessment of parent awareness of child connectedness	.71	5.12 (.95)
Confidence	The level of confidence the worker has in working with families, parents and children	.72	5.71 (1.15)
Need training	Worker willing to undertake further training	.74	5.42 (1.05)
Family support	Providing resources and referral information to consumers and their families	.67	3.91 (1.27)
Referrals	Referring family members to other programmes	.69	4.09 (1.56)

FFPQ subscales, range 0–7

Health professionals were also asked about the number of conversations with parents, the number of conversations with children, and how many parents that had refused conversations with their children during the last 2 months. These were rated; *none *=* 0, one to two *= *1, three to five *=* 2, over five *=* 3.* Health professionals were also asked if they had participated in specific training to deliver family focused practice in accordance with changes to the law. These where rated*; no *=* 0, to some degree *=* 1, yes *=* 2.*

The participants were asked whether their unit had made improvements to better support children while visiting their parents, like a better play area or family room.

During the recruitment process for the larger part of the CHIP-study, 594 registration forms were collected, with anonymous data of the number of patients’ children available for recruitment, controlling whether children were documented in patients’ health records, as required.

### Analysis

Descriptive statistics for characteristics of the participants were calculated and differences between hospitals explored (Table 1). Mean and standard deviations for each of the ten FFPQ subscales with acceptable reliability was calculated (Table 2). A two-way between groups analysis of covariance (ANCOVA) was performed to determine differences between hospitals at the level of family focused practice, controlling for the demographics, professional background, role (CRP and C), and having received specific training (Table 3). As there were no statistically significant interaction effects between the role of personnel and hospitals on any of the subscales, only differences among hospitals are reported. The hospitals also were compared on other aspects of family focused practice. ANOVA was used to calculate differences between hospitals in establishing play areas and family rooms. The number of patient’s children found in the registration forms at the recruitment days, were compared with documentation of patient’s children (in the patient’s electronic health record) and descriptive statistics were used to calculate differences between hospitals and types of services.

**Table 3:** Mean differences (ANCOVA) of Family Focused Practice on Hospitals, adjusted for demographics, professional background, role (CRP or C) and specific training (N = 280)

	H1 (*n* = 73)	H2 (*n* = 41)	H3 (*n* = 43)	H4 (*n *= 65)	H5 (*n* = 58)	df	*F*	eff. size	Sig. *p*	Hospital differences
	*Mean* 95% CI	*Mean* 95% CI	*Mean* 95% CI	*Mean* 95% CI	*Mean* 95% CI					
Organisation										
Workplace support	4.02 3.65–4.40	4.35 3.83–4.87	4.57 4.08–5.06	4.78 4.39–5.17	4.76 4.35–5.18	4.235|	2.463	.04	.046*	H4, H5 > H1
Co-worker support	4.86 4.61–5.16	4.77 4.39–5.44	5.07 4.71–5.44	5.20 4.91–5.49	5.20 4.90–5.51	4.249	1.298		.271	
Time family work	4.53 4.21–4.86	4.42 3.95–4.89	3.95 3.51–4.39	4.74 4.39–5.08	4.47 4.10–4.84	4.246	1.964		.101	
Service available	4.56 4.25–4.86	5.09 4.67–5.51	4.92 4.51–5.33	4.90 4.58–5.22	4.88 4.54–5.22	4.248	1.284		.277	
Worker										
Knowledge skills	4.63 4.41–4.84	4.83 4.53–5.13	4.96 4.67–5.24	5.10 4.87–5.32	5.09 4.87–5.32	4.249	2.943	.05	.021*	H4, H5 > H1
Connectedness	5.04 4.83–5.26	4.92 4.62–5.22	4.96 4.67–5.26	5.29 5.06–5.52	5.27 5.02–5.51	4.250	1.607		.173	
Confidence	5.34 5.27–5.80	5.61 5.23–5.99	5.62 5.26–5.97	6.01 5.72–6.29	5.62 5.33–5.92	4.249	1.613		.171	
Need training	5.68 5.44–5.92	5.40 5.04–5.75	5.35 4.92–5.58	5.19 4.94–5.45	5.54 5.27–5.81	4.243	2.352			
Practice										
Family support	3.81 3.54–4.08	3.63 3.20–4.05	3.32 2.96–3.68	4.03 3.73–4.32	4.27 3.96–4.58	4.218	4.393	.08	.002*	H5 > H1, H2, H3 H1, H4 > H3
Referrals	4.01 3.63–4.39	4.21 3.68–4.74	3.85 3.37–4.33	4.27 3.87–4.68	4.18 3.77–4.58	4.208	.549		.700	
Additional questions										
Conversation parents	1.37 1.15–1.60	1.46 1.15–1.77	1.56 1.26–1.87	1.81 1.56–2.05	1.80 1.56–2.05	4.258	2.378		.052	
Conversations children	.36 .21–.52	.32 .11–.54	.24 .03–.45	.45 .28–.61	.40 .22–.57	4.258	.633		.639	
Parents refused conversation children	.29 .13–.45	− 38 .16–.60	.56 .34–.77	.36 .19–.53	.70 .52–88	4.258	3.249	.05	.011*	H5 > H1, H2, H4

FFPQ subscales, range 1–7, Additional questions, range 0–3, * *p* < .05

## Results

### Descriptive statistics

The highest ratings by the total group of health professionals (*N *= 280) were given on the confidence subscale (*M* = 5.71, range 0–7), in which they stated that they were confident in working with families (Table 2). Nevertheless, they wanted more training (*M* = 5. 42). The lowest total ratings were given on the family support subscale (*M* = 3.91), as when they delivered family support or referred families/children to other services (*M* = 4.09). They agreed to some extent that they had time to work with families (*M* = 4.50).

### Differences of family focused practice on hospitals

Table 3 shows significant differences when the hospitals were compared on *workplace support*, *knowledge and skills, family support.* The ANCOVA analyses controlled for demographics, professional background, role (CRP or C) and specific training. There were also significant differences with respect to age, length of experience, professional background, role, and specific training.

Post Hoc Bonferroni analyses showed that H1 scored significantly lower on *workplace support* than H4 and H5. Significant other differences were found for; specific training and professional background, with social workers receiving less support than nurses. On *knowledge and skills,* H1 scored significantly lower than H4 and H5. Other significant effects were age, having received specific training and role, with child responsible personnel having more knowledge than other clinicians. On *family support*, H5 scored significantly higher than H1, H2 and H3, and H1 and H4 scored significantly higher than H3. Significant effects were also found for specific training and professional background, with social workers delivering more family support than nurses, and psychologists delivering less family support than nurses. *On parents refusing conversations with children,* H5 had significantly more refusals than H1, H2 and H4. Other significant effects were age and received specific training, with younger and less trained health professionals receiving more refusals to include children in the conversations.

### Comparing hospitals on other aspects of family focused practice

As reported by Skogøy [44], the hospitals had to some degree improved the support for children visiting parents at the hospital, e.g. establishing family rooms, establishing play areas or improving routines for welcoming children. We found no significant differences between the hospitals on these variables.

All hospitals had made changes in their data systems to register if the patients had minor children (0–18 years) [44]. Overall, 61% (1540 of 2529 children) were registered in the patient’s health record. However, there were differences between somatic clinics (51%), mental health clinics (61%), and substance abuse clinics (71%) [44]. There were also differences between the hospitals (51–82%), with the highest registrations at the two university hospitals (H3, 77% and H5, 82%) [44], which also scored highest on giving specific training in registration procedures. H4 also had high registration rates in mental health clinics (80%), and substance abuse clinics (69%), but the somatic clinics (cancer and neurology), registered none of the children.

## Discussion

This study examined the type and extent of family focused practice in five Norwegian hospitals following changes to the law and explored differences between the five hospitals. Overall, health professionals in Norway had high knowledge and confidence in working with families and children. However, they showed moderate family support and made few referrals, indicating that the hospitals are still in the installation stage of the policy changes. When the five hospitals were compared, there were significant differences on three family focused practice subscales: workplace support, knowledge and skills, and family support. In addition, there were differences in how many parents refused to have conversations with their children.

### Differences of family focused practice on hospitals

Norwegian health professionals gave high ratings on knowledge and skills, and connectedness, with the highest rating on the confidence scale. This is encouraging and contrasts earlier studies where mental health professionals have been found to lack enough knowledge and skills on how to support patients’ children [30, 55]. However, the implementation of family focused practice was still moderate, with the lowest ratings on family support and referrals. Compared to a study of Australian and Irish psychiatric nurses [27], the health professionals in Norwegian hospitals are more confident in working with families and children than are Australian and Irish psychiatric nurses. However, they score lower than Australian psychiatric nurses on both family support and referrals, while scores on the time for family work, service available and need training subscales are quite similar. Though knowledge and skills, and confidence are high in Norway, this has not yet led to increased family support and referrals for the total group, which indicate that Norwegian hospitals are still in an initial implementation stage of the policy changes

However, there were significant differences between the hospitals on three of the ten FFP-subscales; workplace support, knowledge and skills, and family support. Hospital 1 scored significantly lower than H4 and H5 on workplace support and on knowledge and skills, suggesting that the quality of training and supervision has been poorer at H1. This is supported by earlier findings [44], with this hospital lacking a hospital coordinator, and having the lowest implementation scores, especially on leadership, decision support data systems and supervision.

Despite these lower results on workplace support and knowledge and skills, Hospital 1 scored medium on family support, being significantly higher than H3. It is notable that both professional background and having received specific training also had significant effects on the level of family support, with social workers and nurses giving more family support than psychologists. These results confirm earlier studies showing that family support can be influenced by both organisational and worker-related factors [32, 56].

We expected that H3, with high implementation scores [44], would have scored higher on family support. However, one explanation might be the less time for family work at H3, as time has been identified in earlier studies as predictor of family support [56]. Another explanation might be that the clinicians relay on other services available, with this area having a Next of kin Centre [57]. There are some research showing that working in a rural area can predict FFP [58], suggesting that if there is a lack of other services available, the health professionals might try to support the families. However, these suggestion needs to be further explored, and there might be other important explanations of these differences.

Another notable finding was that both H4 and H5 scored high on having conversations with parents, and giving family support, but H4 had significantly fewer parents who refused to have conversations with children, compared to H5. Barriers to parents’ and children’s’, willingness to take part in conversations have been identified in earlier studies [21, 30]. However, the timing for the conversations might be important [21], if they were planned when patients felt overwhelmed, and needed time to adjust to a severe diagnosis. The patient and their next of kin may also have different needs, as when patient needed treatment and rest, and their next of kin needed information and family support [59]. Family related development projects at H4 have highlighted the importance of health professionals being able to build a trustful relationship in which patients worries and the children’s situation can be discussed [59].

The findings highlight the importance of understanding why parents refuse to have conversations with their children, and whether this is related to health professionals’ attitude, knowledge and skills, supervision, profession or other factors.

### Comparing hospitals on other aspects of family focused practice

Improved routines for children to visit their sick parents were a positive finding in all hospitals included in the study and have been recommended in guidelines for oncology [17], and mental health [30, 60]. Family friendly visiting facilities may also give health professionals more possibilities to interact with children and enable family focused practice [30, 60, 61].

Registration of children in the parent’s health record were considerably higher (61%) than the 44% found in 2012 (only one mental hospital included) [62]. This suggests that in contrast to being described as “hidden children” [63], children have become more visible as next of kin. However, the differences between hospitals (51–82%), and types of services (51–71%) signal that there is room for improvement. Internationally, identification of the parents as consumers of health services, along with their children, is thought to be a key step to integrating a family focused approach [7, 64]. As international estimates of children affected by parental illness vary, high registration rates could give more precise information regarding patients as parents, and the number of children potentially in need of support.

### Implementation stages of family focused practice

Implementation of new practice may conflict with other demands in the hospital, which can affect organisational readiness for change [41]. In the installation stage, all implementation team members should be trained and gain a shared understanding of the intervention and of their implementation role [35, 65]. However, if a hospital has not fully addressed all aspects of the installation stage, e.g. established leadership/implementation teams, secured supervision or established data support systems, these weaknesses could affect the implementation of the next stage. This seems to be the situation at H1, where health professionals scored significantly lower than the other hospitals on workplace support and on knowledge and skills. This is not surprising, as this hospital was found to s score significantly lower than other hospitals on implementation drivers [44], especially on the subscales leadership, decision support data system and supervision.

All hospitals in this study seem to still be in an initial implementation stage, in which they are beginning to deliver new services to families and children. At this stage, it is critical to collect data to determine whether the interventions are being delivered as intended [37]. There were differences between hospitals on registration of children in patient’s health record, family support, and parents refusing conversations with children, which highlight the need for hospitals to use data to determine how to target their improvement strategies. Though health professionals have high knowledge and confidence in their ability to support families, other barriers like lack of workplace support, time and workload constrains or lack of co-worker support and supervision might hinder new family practice behaviour. There is still some time before implementation of the new regulation reaches the full implementation stage, where family focused practice is integrated into usual practice, and it takes time to be able to measure if the intervention leads to long term outcome effects for families [20], and lower societal costs [4].

### Recommendations

Some important recommendations regarding policy, practice and research can be made from the findings of this study. They are as follows:

#### Policy

The findings highlight the need to establish national quality indicators in relation to the law changes, and these could include (a) number of patients registered as parents (b) the registration of children in parents’ health record, suggested by BarnsBeste (Children’s Best Interests)—National Competence Network for Children as Next of Kin in Norway.

The high risk and societal cost of children with parental illness [4] also make it important to discuss whether enough resources have been deployed to establish the preventive efforts stated in the new law. To achieve better results more quickly, special implementation teams are recommended [66, 67].

#### Practice

It is important to tailor improvement strategies to the situation at the hospitals and the specific services. Performance assessment and data systems are found to be important to support implementation of new practices [35, 65]. Creating a structure for implementation, ongoing implementation support strategies and process evaluation, with supportive feedback mechanisms and learning from experience are critical aspects of implementation, as highlighted in a summary of different frameworks and models [68].

#### Research

There is a need to further define the concept of family focused practice, and how this can be measured. Especially, it would be useful to include more detailed questions regarding conversations with parents, parents and children together, and conversations with children alone. It could also be useful to differentiate between knowledge and skills in measurements, as knowledge alone does not necessarily lead to a change of practice.

### Strengths and limitations

The two groups of personnel were recruited from stratified, randomly selected units -from mental health, substance abuse and physical health. This is a key strength of the study. The response rate for child responsible personnel was high (73%). Lower, however (52%) for the sample of clinicians reponsible for the treatment of patients recruited for the larger part of the study. One reason for the lower response rate, was that the second group was recruited via their patients who consented to participate in the larger part of the study. This might have given a recruitment bias, with lower participation from health professionals with less interest in this topic, or with a higher workload (e.g. psychologists and psychicians).

Another limitation was that the family focused practice data relied on personnel self-reports, which might potentially be biased. However, the objective outcome data of children documented in patients’ health records was a strength. This is in line with the recommendation [69, 46] to include other outcomes, like adoption and penetration within an organisation.

A strength of this study is that this measure has been used in other countries, which enables comparisons in both use of the measure and outcomes.

## Conclusion

Overall, health professionals in Norway reported high levels of knowledge and confidence in working with families and children, but the reports on their ability to support family and make referrals were more modest. There were clear differences between hospitals on key variables like workplace support, knowledge and skills, family support and parents refusing conversations with children. The differences highlight the need for leadership to actively follow implementation progress in real time, and to tailor improvement strategies to hospital-specific needs. The findings allowed several recommendations for future policy, practice and research.

## Abbreviations

AIF: Active Implementation Framework
CHIP-study: children of ill parents multicentre study
C: clinicians
CRP: child responsible personnel
FFP: family focused practice
FFPQ: family focused practice questionnaire
H-CRP: hospital coordinators
H: hospital
